# Enzyme-Assisted Extraction of Ulvan from the Green Macroalgae *Ulva fenestrata*

**DOI:** 10.3390/molecules28196781

**Published:** 2023-09-23

**Authors:** Ana Malvis Romero, José Julián Picado Morales, Leon Klose, Andreas Liese

**Affiliations:** Institute of Technical Biocatalysis, Hamburg University of Technology, Denickestraße 15, 21073 Hamburg, Germany

**Keywords:** ulvan, biopolymers, biomaterials, macroalgae, *Ulva fenestrata*

## Abstract

Ulvan is a sulfated polysaccharide extracted from green macroalgae with unique structural and compositional properties. Due to its biocompatibility, biodegradability, and film-forming properties, as well as high stability, ulvan has shown promising potential as an ingredient of biopolymer films such as sustainable and readily biodegradable biomaterials that could replace petroleum-based plastics in diverse applications such as packaging. This work investigates the potential of *Ulva fenestrata* as a source of ulvan. Enzyme-assisted extraction with commercial cellulases (Viscozyme L and Cellulysin) and proteases (Neutrase 0.8L and Flavourzyme) was used for cell wall disruption, and the effect of the extraction time (3, 6, 17, and 20 h) on the ulvan yield and its main characteristics (molecular weight, functional groups, purity, and antioxidant capacity) were investigated. Furthermore, a combined process based on enzymatic and ultrasound extraction was performed. Results showed that higher extraction times led to higher ulvan yields, reaching a maximum of 14.1% dw with Cellulysin after 20 h. The combination of enzymatic and ultrasound-assisted extraction resulted in the highest ulvan extraction (17.9% dw). The relatively high protein content in *U. fenestrata* (19.8% dw) makes the residual biomass, after ulvan extraction, a potential protein source in food and feed applications.

## 1. Introduction

Macroalgae represent a sustainable, unlimited, and almost entirely untapped feedstock for biobased products and could, therefore, play one of the key roles in the desired transition from an economy based on petrochemical products towards a circular and sustainable bioeconomy [1]. A wide range of high-value-added products such as food, feed, nutraceuticals, and fertilizers can be obtained from macroalgae biomass, thereby contributing to the success of the European Blue Economy [2]. Among these compounds, macroalgae biopolymers are characterized by their biocompatibility, biodegradability, film-forming properties, and high stability, which make them potential ingredients for the development of biopolymer films. These are made of natural compounds such as proteins, polysaccharides, and lipids, and represent environmental-friendly alternatives to synthetic plastics [3,4].

Green macroalgae species of the genus Ulva can be found in enormous amounts almost all over the world. These algae are rich in ulvan, a sulfated polysaccharide with a high potential to replace synthetic polymers in the packaging industry due to its great biodegradability, biological activities, and tunable physicochemical and rheological properties [5]. Some studies have proven its potential as a constituent of biopolymer films [6,7,8,9,10]. Nevertheless, to gain access to ulvan, the development and optimization of eco-friendly and cost-effective extraction processes is necessary. The importance of the extraction method and process conditions (e.g., temperature, extraction time, pH, etc.) is due to their direct effect on the yield and physicochemical characteristics of the extracted ulvan and, therefore, on its application in the development of biopolymer films. Guidara et al. [6] extracted ulvan from *Ulva lactuca* by enzymatic chemical extraction and acid extraction, demonstrating that the variation in the extraction conditions had a direct impact on the surface charge and glass transition temperature of the extracted ulvans. Optical, thermal, structural, and antioxidant properties of the formed biofilms were also affected by the extraction conditions, showing that the incorporation of the enzymatically extracted ulvans into the biopolymer films had a positive effect on those properties. Yaich et al. [11] demonstrated that acid extraction and enzymatic–chemical extraction are able to maintain ulvan structure; nevertheless, a significant influence of the extraction method was observed on the thermostability, molecular weight, and antioxidant activity.

Hot water, acid/alkaline, and organic solvent extraction represent the most commonly used methods to extract polymers from macroalgae biomass. However, these can entail significant energy consumption and the use of hazardous chemicals which could lead to adverse environmental consequences. Enzyme-assisted extraction (EAE) of ulvan is not as well investigated as other extraction processes. Nevertheless, some studies have successfully applied EAE to obtain ulvan from different Ulva species such as *Ulva* sp. [12] and *Ulva lactuca* [11,13]. EAE represents an effective and nontoxic procedure with high selectivity, low energy requirements, and gentle conditions [14]. Furthermore, it allows the selective extraction of different macroalgae compounds, ensuring full usage of the biomass and generating few residues through a succession of steps, leading to a biorefinery concept [15]. Therefore, the use of biocatalysis to enhance the extraction of compounds from biological raw material represents a key element in the achievement of the Sustainable Development Goals (SDGs) regarding responsible consumption and production (#12) and life below water (#14) [16].

In this work, the green macroalgae *Ulva fenestrata* was investigated as a source of ulvan. Enzyme-assisted extraction was used for cell wall disruption, and the effect of the enzyme type and extraction time (3, 6, 17, and 20 h) on ulvan yields and its main physicochemical characteristics were investigated. In order to enhance ulvan extraction yields, a combined process based on enzymatic and ultrasound extraction was performed. Among the enzymes used, Cellulysin treatment led to the highest ulvan yield (14.1% dw). Furthermore, the combination of ultrasound and enzyme extraction resulted in a yield improvement of 49.6 and 51.7% compared to single enzymatic and ultrasound extractions, respectively.

## 2. Results

### 2.1. Ulva fenestrata Biochemical Composition

**Figure 1:**
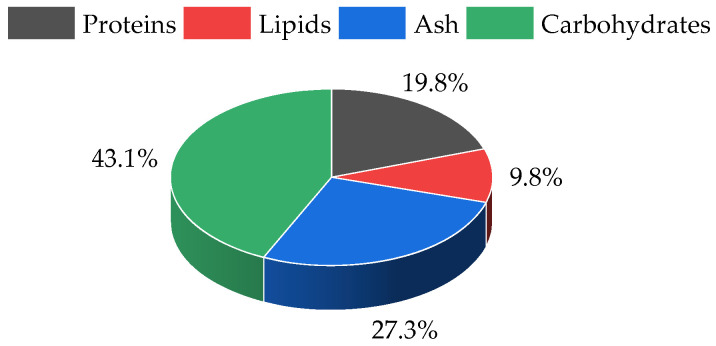
Biochemical composition of *Ulva fenestrata* biomass.

*U. fenestrata* contained 19.8 ± 1.31% proteins, 9.79 ± 0.003% lipids and 27.3 ± 0.25% ash (Figure 1). The total carbohydrate content (43.1% dw) was calculated considering that proteins, lipids, ashes, and carbohydrates are the main macroalgae biomass components and account for 100% dw. 

The respective content of each metabolite in macroalgae is highly variable depending on the season, geographical location, state of growth, nutrients availability, etc. As proven by Steinhagen et al. [17], the protein content in *U. fenestrata* is higher in early spring (April), while carbohydrates increase later in the season (May–June). Carbohydrates and proteins from macroalgae represent high-added-value compounds with numerous potential applications such as the formation of biopolymer films from carbohydrates and the use of proteins in food and feed. 

*U. fenestrata* composition confirmed that polysaccharides are the main constituents of the biomass, where they function as structural and storage blocks. Olsson et al. [18] showed that temperature, nitrogen level, and pCO_2_ are the main cultivation parameters affecting the total amount of carbohydrates in *U. fenestrata.* The total carbohydrate content of the biomass used in this work is in the middle to higher range of what has been reported for Ulva species (15–65% dw). This fraction is composed of glucose, rhamnose, xylose, glucuronic acid, iduronic acid, and galactose [19]. 

Protein content also varies significantly depending on environmental conditions and nutrient availability, which is why reported values in the literature can range from 10 to 26% dw [20]. The importance of the protein content is due to the potential use of *Ulva* spp. as a new and sustainable protein source, with a protein production potential competitive with those produced from soybean. In addition, the extraction of ulvan could result in a high yield of protein-enriched ‘’residual biomass’’, as demonstrated by Magnusson et al. [21].

Not only the amount of protein but also the protein quality, determined as the essential amino acids content (EAA), is important. Table 1 shows the amino acid profile of *Ulva fenestrata* compared to the World Health Organization (WHO) recommendations for the relative distribution of essential amino acids in foods. Results show that *U. fenestrata* contains the full profile of essential amino acids (with a relative high content of 38.9% EAA) and meet or exceed the WHO recommendations. Therefore, this species represents a potential source of plant-based proteins.

### 2.2. Ulvan Extraction

#### 2.2.1. Enzyme-Assisted Extraction: Effect of Extraction Time

The effect of the extraction time (3, 6, 17, and 20 h) on ulvan extraction yields was studied for the cellulase blends (Viscozyme L and Cellulysin) and proteases (Neutrase 0.8L and Flavourzyme). The same enzyme activity (300 U g^−1^_Biomass_) was used in all reactions. To provide better contact of the enzymes with the biomass, constant stirring of 150 min^−1^ was applied in all reactions with a Köttermann 2737 incubator (Köttermann GmbH, Germany).

**Figure 2:**
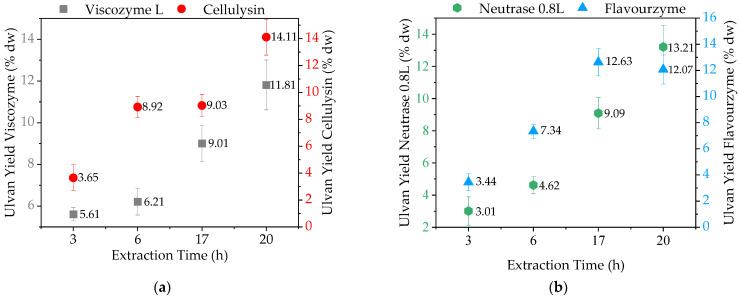
Ulvan extraction yields in dependency of extraction time: (**a**) Cellulases: Viscozyme L (5 g biomass, 100 mL 0.1 M NaOAc, pH 5, 300 U g^−1^_Biomass_, 50 °C, 150 min^−1^) and Cellulysin (5 g biomass, 100 mL 0.1 M NaOAc, pH 5, 300 U g^−1^, 40 °C, 150 min^−1^); (**b**) Proteases: Neutrase 0.8L (5 g biomass, 100 mL 0.1 M Tris HCl, pH 7, 300 U g^−1^_Biomass_, 60 °C, 150 min^−1^) and Flavourzyme (5 g biomass, 100 mL 0.1 M Tris HCl, pH 7, 300 U g^−1^_Biomass_, 50 °C, 150 min^−1^).

As observed in Figure 2, the extraction time had a strong effect on ulvan yields. Increasing the time from 3 to 20 h led to an ulvan extraction improvement which ranged from 52.5% (Viscozyme L) to 77.2% (Neutrase 0.8L). For both cellulases (Figure 2a), higher extraction times led to higher yields, reaching a maximum of 14.11% with Cellulysin after 20 h of extraction. This might be associated with the composition of both enzyme preparations. While Viscozyme L primarily harbors cellulase, xylanase, and pectinase activity, Cellulysin is a β-glucosidase with a higher specificity for β-1,4-glycosidic and β-1,3 bonds (found in internal polysaccharide linkages) compared to Viscozyme L. This might result in higher yields and purity of the extracted ulvan [22,23]. Furthermore, the optimal reaction temperature for Cellulysin is lower than that for Viscozyme L, which is less likely to damage the ulvan and can result in better yields, as also shown by the molecular weight distribution results (see Section 2.3.1).

Regarding the proteases (Figure 2b), a maximum yield of 13.21% was obtained with Neutrase 0.8L after 20 h of reaction. A slightly different behavior was observed with Flavourzyme, which led to the highest yield (12.63%) after 17 h. This might be due to a faster enzyme deactivation. Eberhardt et al. [24] showed that Flavourzyme is more sensible to inactivation when used for hydrolytic reactions in comparison to other commercial proteolytic enzymes, like Alcalase. A similar behavior was observed by Rosa et al. [25], who evaluated four commercial proteases in terms of the hydrolysis degree and showed that, while Alcalase was able to remain active for 15 min in a 90 min reaction, Flavourzyme was only active for 10 min. This might be related to the composition of both enzyme blends. Flavourzyme is a mixture of several different proteases (trypsin, chymotrypsin, peptidase, etc.) with a more limited pH stability range compared to Neutrase 0.8L, which is stable over a wide range of pH values ranging from 5 to 12. A change in the pH value during ulvan EAE could have led to faster deactivation of Flavourzyme. 

Guidara et al. [6] achieved a maximum ulvan yield of 17.95% dw from *Ulva lactuca* when using hot water in the presence of a cellulase and a protease for 2 h. The higher yield in a lower time could be explained by the combined effect of both enzymes types and the hot water, which enhances ulvan extraction. In the study performed by Chen et al. [26], 25.3% of ulvan was extracted from *Ulva pertusa* by using a cellulase for 2.5 h. In contrast with this work, in which the specific activity was 300 U g^−1^_Biomass_, the enzyme activity used by Chen et al. [26] was 2500 U g^−1^_Biomass_, which would explain the increase in hydrolysis with the shorter time. This study demonstrates that increasing the enzyme activity can result in an improvement of the process by increasing the ulvan extraction yields and reducing extraction times. Yaich et al. [11] achieved the highest ulvan yields (17.14% dw) by enzymatic extraction with hot water in the presence of a cellulase and a protease. The characterization of the extracted ulvans revealed that while ulvan structure and properties were maintained, the antioxidant activity was reduced compared to acidic extraction. Wahlström et al. [27] achieved an ulvan yield of 11 ± 3% dw with ethanol treatment and hot water followed by enzymatic purification using an α-amylase (20 U g^−1^_Biomass_, 20 °C, 1 h) followed by a proteinase K (6.4 U g^−1^_Biomass_, 37 °C, 24 h). These ulvans were purer and the structure was less altered (as deduced from the lower starch content and higher sulfation degree) than those extracted by hydrochloric acid. The application of two pretreatment steps (ethanol wash and hot water) prior to enzymatic hydrolysis could explain the lower relative enzyme activity used compared to this study. 

#### 2.2.2. Combined Enzymatic and Ultrasound Extraction

**Figure 3:**
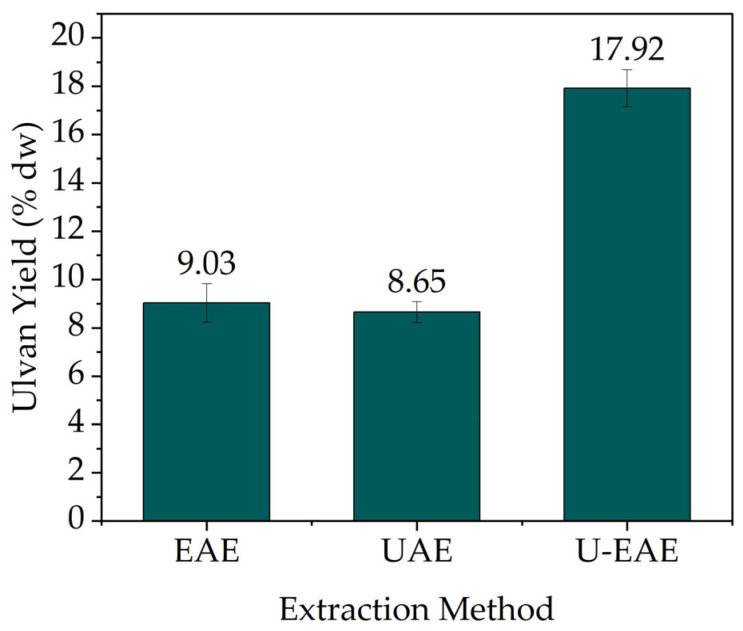
Ulvan extraction yields with enzyme-assisted extraction (EAE), ultrasound-assisted extraction (UAE), and combined ultrasound with enzymatic extraction (EAE: 5 g biomass, 100 mL 0.1 M NaOAc, pH 5, 300 U g^−1^_Biomass_ Cellulysin, 40 °C, 150 min^−1^, 17 h, UAE: 80% amplitude, 40 min).

Mechanical extraction methods are the most commonly used for ulvan extraction. Among these, ultrasound-assisted extraction (UAE) offers several advantages such as high yields, short extraction times, lower energy input, and absence of organic solvents or harsh chemicals [28,29]. Therefore, ultrasound-assisted extraction was applied (80% amplitude signal, 40 min) following a 17 h enzymatic extraction with Cellulysin (U-EAE) as well as the single extraction method (UAE) (Figure 3). 

As seen in Figure 3, the combination of ultrasound with enzyme extraction led to the highest ulvan extraction yield (17.92% dw), resulting in a yield improvement of 49.6 and 51.7% when comparing U-EAE with single EAE and UAE, respectively. The use of cell-wall-degrading enzymes such as Cellulysin can efficiently release polysaccharides from the cell wall matrix, increasing ulvan yields. In UAE, the ultrasonic vibrations create cavities in the cell wall, promoting cell wall disruption and the release of ulvan [30]. Therefore, the combination of these two methods constitutes an effective approach which does not require the use of harsh chemicals or solvents. The effectiveness of UAE in improving the extraction efficiency of ulvan from *U. pertusa* (20.6%) [26] and *U. lactuca* (17.6%) [31] was demonstrated.

### 2.3. Characterization of Extracted Ulvan

#### 2.3.1. Molecular Weight Distribution

The molecular weight distribution (M_w_) of all extracted ulvans was assessed by gel permeation chromatography (GPC) in order to determine the effect of the extraction method on the size of ulvan polymers. 

**Figure 4:**
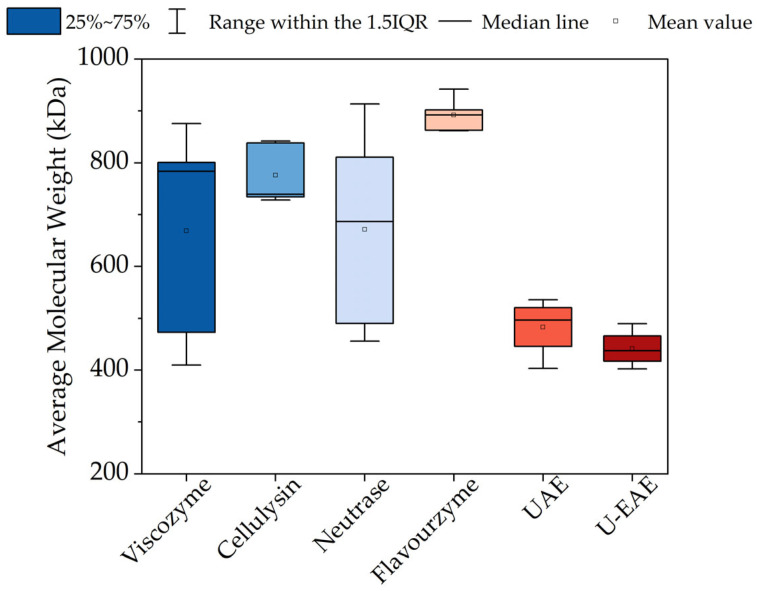
Box plots of the average molecular weight (M_w_) values of all extracted ulvans determined by gel permeation chromatography. IQR, interquartile range.

As shown in Figure 4, a wide range of molecular weight values were registered, ranging from 402 kDa (U-EAE) to 942 kDa (Flavourzyme, 3 h extraction). Results show that the application of ultrasound (UAE and U-EAE) led to a significant decrease in the molecular weight distribution compared to enzyme-assisted extraction, which could be due to higher ulvan depolymerization due to the application of mechanical force.

This high heterogeneity in the ulvan size and the influence of the extraction method has been described in the literature. Other parameters influencing the molecular weight of ulvan include the source, species, extraction conditions, degree of sulfation and ramification, monosaccharide composition, etc. Amor et al. [32] reported M_w_ for ulvan extracted from *Ulva* sp. ranging from 201.1 to 1841 kDa depending on the extraction method and time, concluding that grinding and maceration lead to smaller ulvan fractions compared to Soxhlet extraction. Kazemi et al. [33] also proved the influence of the extraction method on ulvan M_w._ Acid and alkaline extraction led to lower molecular weight (88 and 110 kDa, respectively) in comparison with hot water extraction (300 kDa), suggesting that the ulvan chains were degraded during acid and alkali extraction. 

The importance of the molecular weight distribution is due to its direct link with ulvan physicochemical properties and biological activities and, therefore, on the biopolymer film properties. In general, high-molecular-weight ulvan is preferable for biopolymer films due to its better film-forming properties such as improved gelling, mechanical, and barrier properties. This can lead to a better performance of the films such as higher mechanical performance and water resistance [7,8]. As observed in Figure 4, Flavourzyme resulted not only in the highest M_w_ values (892 kDa on average) but also in a very narrow distribution in the extracted ulvans. This indicates more homogeneous ulvan fractions with similar molecular weights. This homogeneity could contribute to the mechanical properties and stability of the films since a homogeneous molecular weight distribution is more likely to form stable films since the interactions between the polymer chains are more predictable and lead to a more uniform structure [32]. 

#### 2.3.2. Purity and Total Antioxidant Capacity

The extent of the purity of the ulvan fractions was determined in terms of their total phenolic compounds (TPCs) and proteins content. Ulvan is mainly composed of rhamnose, uronic acid, and sulfated xylose and does not naturally contain proteins and phenolic compounds. Nevertheless, during the extraction and purification of ulvan, small amounts of these compounds may be present because they are part of the structure of cell walls closely associated with polysaccharides.

Results showed that the use of different enzymes and extraction times did not have a significant influence on the TPCs content (Table 2). Among all enzymes, Viscozyme L led to the highest TPCs content (0.25 ± 0.024 g kg^−1^). On the contrary, the protein content varied significantly depending on the enzyme used: the use of proteases resulted in a higher protein coextraction (24.5 ± 4.50 and 62.5 ± 7.05 g kg^−1^ for Viscozyme L and Cellulysin, respectively), resulting in ulvans containing an average of 13 and 8.15% dw of proteins (Neutrase 0.8L and Flavourzyme, respectively), compared to 2.45 and 6.25% dw in the ulvan extracted by cellulases (Viscozyme L and Cellulysin, respectively). 

These values are comparable with those described in the literature. Yaich et al. [11] and Ibrahim et al. [34] registered protein contents of 3.57 and 9.67% dw, respectively, in ulvan from *U. lactuca.* Robic et al. [35] reported protein values of 7.1–22% in ulvan isolated from *U. rotundata* and 10.9–16.8% from *U. armorican.* Ulvan extracted from *U. ohnoi* contained proteins between 0.4 and 5.9% after hydrochloric acid and sodium oxalate extraction, respectively [36]. 

Regarding the total antioxidant capacity (TAC), results showed that this was not negatively affected by the type of enzyme or extraction time used, resulting in the highest average TAC 197 ± 94.5 g kg^−1^ when using Cellulysin. Ulvan from green macroalgae has been reported to possess a great antioxidant activity, which makes it a potential ingredient of food wrappings and films [5]. Different studies have shown the potential of ulvan in the production of edible films with ulvan as natural antioxidant agent that can effectively prevent food oxidation and the formation of undesirable flavors [8,37].

**Figure 5:**
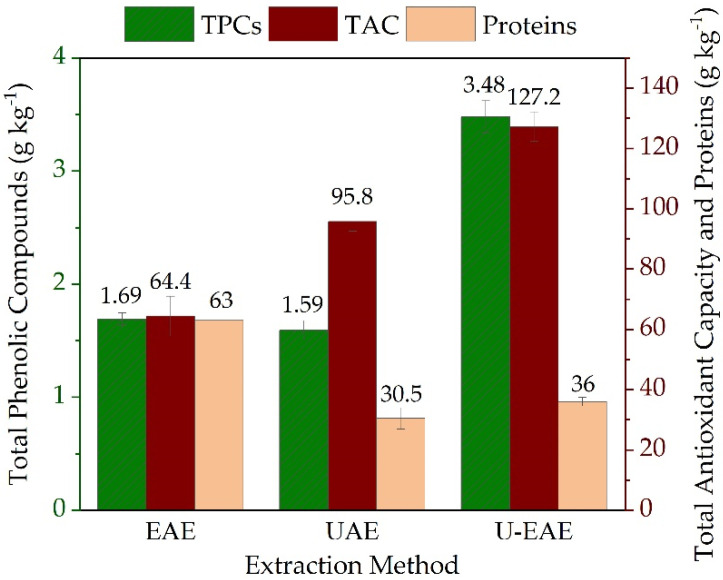
Quantification of total phenolic compounds (TPCs), proteins, and total antioxidant capacity (TAC) based on ulvan extracted by EAE and combined U-EAE (EAE: 5 g biomass, 100 mL 0.1 M NaOAc, pH 5, 300 U g^−1^_Biomass_ Cellulysin, 40 °C, 150 min^−1^, 17 h, UAE: 80% amplitude, 40 min).

The effect of the extraction method on ulvan purity and TAC was also studied. Figure 5 shows TPCs, proteins content, and TAC in ulvans extracted by enzymatic (EAE), ultrasound (UAE), and combined (U-EAE) processes.

Regarding the phenols content, the use of U-EAE resulted in an increase of 51.3 and 54.1% compared to EAE and UAE, respectively. On the contrary, the protein content experienced a reduction of 51.6 and 57.1% with UAE and U-EAE, respectively. One reason for that could be a higher possibility for protein denaturation when applying ultrasound. Regarding TAC, a notable increase can be observed when using ultrasound on its own or in combination. Enzymatic extraction requires milder conditions compared to mechanical methods like ultrasound. Furthermore, enzymes are very selective and specific to certain bonds, which causes less physical cell wall and ulvan disruption (as observed in Figure 4). Therefore, the application of ultrasound could have led to a higher coextraction of other *U. fenestrata* antioxidant compounds (e.g., flavonoids, carotenoids, ascorbic acid, tocopherols, etc.), resulting in a higher TAC in the extracted ulvan.

Although proteins and phenolic compounds are considered ulvan impurities, it has been shown that their presence can be favorable in the development of ulvan-based biopolymer films. Proteins have film-forming, excellent mechanical and barrier properties. As proven by Chakravartula et al. [38], blending polysaccharides and proteins is an effective approach to improve the properties of edible films. On the other hand, the addition of polyphenols to a polysaccharide film made of pectin and chitosan resulted in a film with higher thickness, water permeability, and antioxidant activity [39].

#### 2.3.3. ATR-FTIR Analysis

The extracted ulvan fractions of the EAE, UAE, and U-EAE treatment were subjected to attenuated total reflection-Fourier transformation infrared spectroscopy (ATR-FTIR) and compared to a commercially available ulvan standard to validate the chemical structure of the extracts and derive potential impurities. 

**Figure 6:**
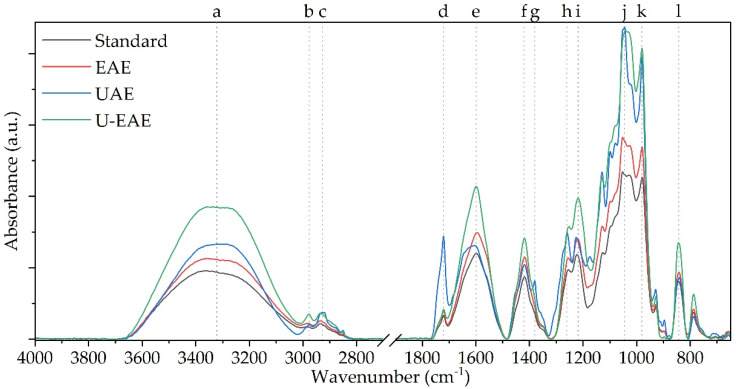
ATR-FTIR spectra of ulvan extracts after EAE, UAE, and U-EAE (EAE: 5 g biomass, 100 mL 0.1 M NaOAc pH 5, 300 U g^−1^_Biomass_ Viscozyme L, 50 °C, 150 min^−1^, UAE: 80% amplitude, 40 min) compared to ulvan standard.

The reference spectrum of ulvan served as the baseline for comparison with the spectra of the extracted ulvan, revealing distinct bands that correspond to specific structural elements within the polymer (Table 3, Figure 6). The peak at 3500–3000 cm^−1^ (a) indicated the presence of hydroxyl groups (–OH) as part of the sugar backbone of the polymer. The peaks at 2970 cm^−1^ (b) and 2930 cm^−1^ (c) were attributed to aliphatic hydrocarbon chains, signifying the presence of C–H stretching vibrations. The peak at 1720 cm^−1^ (d) was assigned to carboxylic acid groups (–COOH) based on C=O stretching vibrations. This finding suggests the presence of uronic and/or iduronic acid residues in the ulvan, which are—among xylose and rhamnose—the main building blocks of the polymer [19]. As ulvan is known to comprise a sulfated backbone, the bands at 1260 cm^−1^ (h) and 840 cm^−1^ (l) belong to the S=O and C–O–S stretching vibrations [34]. The peaks at 1215 cm^−1^ (i), 1045 cm^−1^ (j), and 980 cm^−1^ (k) correspond to C–O stretching vibrations, related to the presence of the glycosidic bonds between the sugar monomers and the acid residues, as described by Ramu Ganesan et al. [8].

The analysis of ulvan extracted by different methods revealed that the main structural elements of the ulvan polymer were present in all cases, indicating successful extraction of an ulvan-rich fraction. However, variations in the spectra were observed among the different extraction methods. The closest match to the reference spectra was found with the enzymatic extraction, which is consistent with the results obtained for impurities expressed as TPC and TAC (see Section 2.3.3). Slight increases in intensity in bands a, e, f, h, and i may be due to the residual protein in the sample of approximately 60 g kg^−1^. A similar band pattern was observed by Chakravartula et al. [38], who blended ulvan with pectin for the preparation of edible composite films. In particular, the amide peaks at 1600–1700 and between 1200–1230 cm^−1^ were strongly pronounced, which is also reflected in the measured spectra.

In comparison to that, both UAE and U-EAE showed stronger deviations from the reference spectra. Particularly, in the fraction obtained after U-EAE treatment, an increase in the intensities of bands a, e, f, h, i, j, k, and l was noted. The observed intensification of these bands could be attributed to the presence and co-extraction of phenolic compounds present in macroalgae, such as gallic acid, epicatechin, rutin, or phlorotannins [40]. As less protein contamination but a much higher TAC was measured in the U-EAE samples compared to EAE (see Figure 5), it is reasonable to assume that these components are responsible for the altered peak intensities. In addition, the increased presence of molecules with antioxidant properties such as flavonoids or carotenoids could have led to an increase in the corresponding bands as well.

### 2.4. Potential Valorization of the Residual Biomass

The sustainable and economical utilization of macroalgae biomass requires the complete valorization of all fractions, thus reducing waste generation. Therefore, the residual biomass after ulvan extraction with Cellulysin (300 U g^−1^_Biomass_, 20 h), Neutrase 0.8L (300 U g^−1^_Biomass_, 20 h) and U-EAE was characterized in terms of its protein content and amino acid composition to determine its potential as alternative protein source (Table 4).

**Figure 7:**
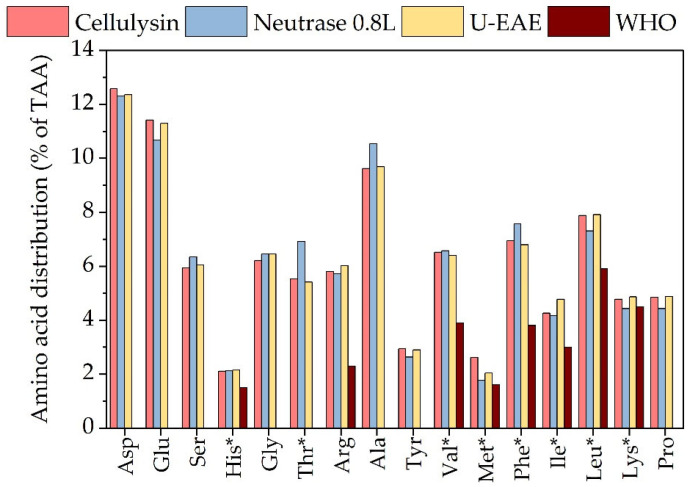
Amino acid profile of the residual biomass after ulvan EAE with Cellulysin (5 g biomass, 100 mL 0.1 M NaOAc, pH 5, 300 U g^−1^_Biomass_, 40 °C, 150 min^−1^, 20 h) and Neutrase 0.8L (5 g biomass, 100 mL 0.1 M Tris HCl, pH 7, 300 U g^−1^_Biomass_, 60 °C, 150 min^−1^, 20 h) and combined ultrasound and enzyme extraction (EAE: 5 g biomass, 100 mL 0.1 M NaOAc, pH 5, 300 U g^−1^_Biomass_ Cellulysin, 40 °C, 150 min^−1^, UAE: 80% amplitude, 40 min). Asp (aspartic acid), Glu (glutamic acid), Ser (serine), His (histidine), Gly (glycine), Thr (threonine), Arg (arginine), Ala (alanine), Tyr (tyrosine), Val (Valine), Met (methionine), Phe (phenylalanine), Ile (isoleucine), Leu (leucine) and Lys (lysine). * Essential amino acids.

As shown in Figure 7, the use of different enzyme types and extraction methods did not have a significant effect on the amino acid distribution of the residual biomass. All samples contained the full amino acid profile and displayed a higher content of essential amino acids than the WHO requirements [17]. The quality of the proteins, determined as the EAA content, ranged from 40.4 (U-EAE) to 40.9% EAA (Neutrase 0.8L), which is in a similar range to other protein sources such as corn meal (41.3 ± 0.3%), rice meal (40.9 ± 0.3%), soy meal (40 ± 0.2%), and wheat meal (30.3 ± 0.2%) [41]. 

Protein content was 26.5, 18.3, and 23.7% dw in the biomass extracted with Cellulysin, Neutrase 0.8L, and U-EAE, respectively (Table 4). As observed in Table 2, the use of Neutrase 0.8L (a protease) led to a larger coextraction of proteins with ulvan, which would explain the lower protein concentration in the residual biomass. Nevertheless, residual biomass from Cellulysin and U-EAE showed a higher protein concentration compared to the initial *Ulva fenestrata* biomass (19.8% dw). This behavior was also observed by Magnusson et al. [21] after extracting salts and ulvan from the green macroalgae *Ulva ohnoi* for the production of high-protein feed and food. The authors showed that increasing protein concentration from 22.2 to 39.5% dw is possible by applying a last enrichment step based on enzyme hydrolysis. Although the protein content in the residual biomass is still low compared to other protein sources such as soy (45–49% dw), the application of a final enrichment step could represent a potential approach to increase the protein content, as shown by Magnusson et al. [21].

## 3. Materials and Methods

### 3.1. Macroalgae Biomass

*Ulva fenestrata* biomass was supplied by the company ALGA+ Ltd. (Ílhavo, Portugal) in the form of washed and dried flakes (product name: Sea-lettuce moi PT-BIO-03). Prior to ulvan extraction, biomass was milled to a particle size of <5 mm in a Retsch ZM 200 mill (Retsch GmbH, Nordrhein-Westfalen, Germany) and stored at room temperature until further use. 

### 3.2. Biomass Biochemical Characterization

Total protein, amino acids distribution, and lipid and ash content were determined from *Ulva fenestrata* biomass, as described below. The total carbohydrate content was calculated considering that protein, lipid, ash, and carbohydrates are the main macroalgae biomass components and account for 100% dw [42]. For all analysis, samples were placed in a −20 °C freezer overnight and then lyophilized on an Alpha 1–2 LD plus freeze dryer (Martin Christ Gefriertrocknungsanlagen GmbH, Germany) in a two-step procedure including main drying and final drying in a laboratory. The pressure on the freeze dryer was set to 0.030 mbar and samples were dried overnight. Finally, samples were grounded to ≤0.5 mm particles. All measurements were performed in duplicate.

#### 3.2.1. Proteins and Amino Acids Distribution

High-performance liquid chromatography (HPLC) was used for total protein determination by quantification of proteinogenic amino acids according to Lamp et al. [43]. The equipment consisted of an Agilent Infinity 1260 HPLC Series (Agilent Technologies, Santa Clara, CA, USA) with fluorescence detector and an LC-Poroshell HPH-C18 separation column (4.6 × 100 mm, 2.7 µm; Agilent Technologies, USA; Part No: 695975-702). The HPLC is operated in gradient mode with a flow rate of 1.5 mL min^−1^ and a column temperature of 40 °C. The mobile phase is operated according to the injection and gradient program described by Lamp [44]. Typical retention times (min) for each amino acid standard are 0.875 (aspartic acid), 1.320 (glutamic acid), 3.471 (serine), 4.208 (histidine), 4.404 (glycine), 4.584 (threonine), 5.294 (arginine), 5.520 (alanine), 6.522 (tyrosine), 7.936 (valine), 8.111 (methionine), 9.086 (phenylalanine), 9.237 (isoleucine), 9.739 (leucine), and 10.158 (lysine). 

Samples were prepared according to Lamp et al. [43]. A total of 0.3 g of biomass was weighed and placed in 100 mL DURAN bottles (DKW Life Sciences, Mainz, Germany) with 25 mL of 6 M HCl solution. The bottles were placed in a UT 6200 ventilated oven (Heraeus, Hanau, Germany) for 24 h at 110 °C. Subsequently, the solutions were placed on ice to stop the hydrolysis. The pH of the solutions was adjusted to pH 1 using 10 M NaOH. Then, the solution was poured into a 200 mL volumetric flask containing 2 mL of each of the protein standards sarcosine and L-norvaline (Merck, Darmstadt, Germany). The volumetric flask was filled to 200 mL using 0.1 M HCl with pH 1. The liquid was then filtered through Agilent PES filters (13 mm diameter and 0.45 µm pore size) into 1.5 mL HPLC flasks. The protein content is expressed as the sum of the mass of amino acids per mass of original biomass.

#### 3.2.2. Lipids

The analysis of the total lipid content was performed according to Ryckebosch and Foubert [45]. For the lipid extraction process, 2 g of biomass were weighed and enclosed within a cellulose extraction thimble, which was then placed within a Soxhlet apparatus. Subsequently, a round-bottom flask was filled with 160 mL of a chloroform and methanol mixture in a 1:1 volumetric ratio, and the mixture was heated to its boiling point. Following 25 extraction cycles, the system was allowed to cool, and 40 mL of deionized water were added. The mixture was shaken for 30 min at the highest agitation rate to facilitate the separation of all nonlipid components into the polar phase. Subsequently, the resulting mixture was transferred into a separatory funnel until complete phase separation was achieved. The chloroform layer was then passed through a funnel equipped with a Whatman No. 1 cellulose filter, containing a layer of anhydrous sodium sulfate to effectively eliminate nonlipid contaminants. The solvent was removed using a rotary evaporator and the round-bottom flask was subjected to 2 h drying at 105 °C within an oven. Following this drying process and subsequent cooling in a desiccator, the total lipid content was determined through gravimetric analysis and expressed as the mass of total lipids per unit mass of the initial macroalgae biomass.

#### 3.2.3. Ash 

The ash content was determined by using a laboratory muffle furnace according to DIN EN ISO 18122 at 550 °C [46]. Samples underwent incineration, followed by a cooling phase inside a desiccator, and were then subjected to gravimetric analysis. Results as expressed as the mass ratio between the heated macroalgae mass and the initial macroalgae mass.

### 3.3. Ulvan Extraction and Purification

#### 3.3.1. Enzyme Activity Assays

For EAE, two commercial cellulase blends (Viscozyme L and Cellulysin) and two commercial proteases (Neutrase 0.8L and Flavourzyme) from Sigma-Aldrich (Darmstadt, Germany) were used. These enzyme formulations were selected based on the composition of *Ulva fenestrata* cell wall, with ulvan and cellulose as main components and intercellularly bounded proteins, among other compounds [47,48]. In all experiments, 300 activity units (U) of each enzyme were used per gram of macroalgae biomass. 

The 3,5-dinitrosalicylic acid (DNS) assay was used to determine cellulase activity. This assay is based on the quantification of released glucose monomers after enzymatic hydrolysis of carboxymethyl cellulose (CMC) [49]. A total of 200 µL of CMC was mixed with 200 µL of 0.1 M sodium acetate buffer, 50 µL of dH_2_O, and 50 µL of Viscozyme L or Cellulysin. After 15 min at 50 °C and pH 5 for Viscozyme L and 40 °C and pH 5 for Cellulysin, the reaction was stopped by placing the tubes on ice for 10 min. Then, 500 µL of DNS reagent were added and the solution was boiled for 10 min in a water bath. After cooling down to room temperature, the absorbance was determined at 546 nm. The cellulase activity was measured based on a glucose standard calibration curve. One unit of activity (U) was defined as the amount of enzyme that releases 1 µmol of glucose within 1 min [50]. 

The azocasein assay was used to determine protease activity. This assay is based on the quantification of released peptides (loaded with the azo dye) after hydrolysis of the protein casein. A total of 140 µL of azocasein solution was mixed with 120 µL of enzyme and incubated at 60 °C and pH 7 for Neutrase 0.8L and 50 °C and pH 5 for Flavourzyme. After 15 min, the reaction was stopped by adding 600 µL of 10 % *w*/*v* trichloroacetic acid. The solution was left on ice for 10 min and centrifuged at 13,000 rpm at 4 °C for 10 min. A total of 800 µL of the supernatant was pipetted into a cuvette and neutralized with 200 µL NaOH (1 M). Absorbance was measured at 420 nm. One unit of activity (U) was defined as the amount of enzyme which yielded an increase in A_420_ of 0.01 [51,52].

#### 3.3.2. Enzyme-Assisted Extraction (EAE)

EAE with cellulase blends was performed in 100 mL of 0.1 M sodium acetate buffer at pH 5 and 50 °C (Viscozyme L) or 40 °C (Cellulysin). For proteases, 100 mL of 0.1 M Tris HCl buffer at pH 7 and 60 °C (Neutrase 0.8L) and pH 5 at 50 °C (Flavourzyme) was used. All experiments were performed in duplicate and included a negative control performed in the same conditions as EAE containing deionized water instead of enzyme. For ulvan EAE, 5 g of milled biomass were incubated with 100 mL of the corresponding buffer at 150 min^−1^ in a Köttermann 2737 incubator (Köttermann GmbH, Uetze, Germany). 

While some studies utilize shorter extraction times, typically ranging from 2 to 5 h, for ulvan enzymatic extraction [26,53], it is worth noting that, given the high complexity of macroalgae cell walls, extended extraction times, of up to 24 h, may prove advantageous in the enzymatic extraction of macroalgae biopolymers [54,55,56]. Consequently, four distinct extraction times were investigated: 3, 6, 17, and 20 h. After the reaction, the enzymatic reaction was stopped by boiling the mixture at 100 °C for 10 min in a water bath.

For ulvan purification, the obtained hydrolysate after EAE was centrifuged (10,000 min^−1^, 4 °C, 20 min) and the supernatant subjected to dead-end ultrafiltration using Amicon stirred cells (Millipore Corp., USA) with 10 kDa molecular weight cut off polyethersulfone membranes. The remaining viscous retentate was collected in a falcon tube and stored at −4 °C. Following the ultrafiltration, a solvent precipitation step was performed by adding 96% (*v*/*v*) ethanol (1:3 biomass/ethanol), and this washing step was repeated three times. Samples were then oven-dried at 40 °C for 3 h to remove ethanol residues, freeze-dried (see Section 3.2), and milled to a particle size of 0.5 mm. Ulvan yields were calculated with respect to the initial dry weight biomass, corrected by substraction of the negative control yield. 

#### 3.3.3. Combined Enzymatic–Ultrasound Extraction (U-EAE) and Ultrasound-Assisted Extraction (UAE)

For combined enzymatic–ultrasound extraction (U-EAE), 5 g of biomass were soaked in 100 mL of 0.1 M sodium acetate buffer with pH 5. Cellulysin was added (300 U g^−1^_Biomass_) and the mixture was incubated at 40 °C for 17 h. After enzymatic hydrolysis, the mixture was sonicated with an HD 2700 ultrasound machine (Bandelin, Berlin, Germany) with 70 W power. The KE 76 ultrasound probe (Bandelin, Germany) was immersed in the solution and the ultrasound settings were fixed to 80% amplitude (20 kHz) for 40 min [57]. 

Ultrasound-assisted extraction (UAE) was performed as described above (U-EAE process conditions) by adding water instead of enzyme to test the sole effect of the sonication in the extraction of ulvan. 

### 3.4. Ulvan Characterization

#### 3.4.1. Gel Permeation Chromatography

Gel permeation chromatography (GPC) was used to assess the molecular weight distribution of all extracted ulvans. The device consisted of two PL aquagel-OH Mixed-H 8 μm 300 × 7.5 mm columns (Agilent Technologies, Santa Clara, CA, USA). For sample preparation, extracted ulvans were diluted in 0.1 M sodium nitrate to a concentration of 1 mg mL^−1^. The solutions were stirred overnight at room temperature to ensure complete solubilization and filtered through a 0.45 µm nylon filter before injection into the GPC. The analysis was conducted at 30 °C and a flow rate of 1 mL min^−1^. Columns were calibrated with a preweighed calibration kit EasiVial comprising polyethylene oxide (PEO) and polyethylene glycol (PEG), (Sigma-Aldrich, Darmstadt, Germany). All measurements were performed in duplicate.

#### 3.4.2. Total Phenolic Compounds

The Folin–Ciocalteau assay was used for the quantification of total phenolic compounds in the extracted ulvans [58]. For sample preparation, 1 g of freeze-dried ulvan was crushed in liquid nitrogen and mixed with 50 mL of 50% (*v*/*v*) methanol. The mixture was subjected to continuous agitation for 1 h within an orbital shaker and filtered through filter paper [59]. Following sample preparation, 20 µL of the extract were mixed with 180 µL of Folin–Ciocalteau’s reagent incubated in darkness for 90 min at room temperature. Absorbance was subsequently measured at 760 nm (ε = 8.3 L mol^−1^ m^−1^) using a UV–Vis spectrophotometer UV-1280 (Shimadzu, Kyoto, Japan). A set of gallic acid standards with concentrations ranging from 10 to 300 µg mL^−1^ was used for calibration. 

#### 3.4.3. Total Antioxidant Capacity

The phosphomolybdate assay was used to determine the total antioxidant capacity of extracted ulvans. Briefly, 0.1 mL of sample was mixed with 3 mL of a TAC solution containing 0.6 M sulfuric acid, 28 mM sodium phosphate, and 4 mM ammonium heptamolybdate. The mixture was then incubated at 95 °C for 90 min, and after cooling to room temperature, the absorbance was measured at 695 nm (ε = 0.3 L mol^−1^ m^−1^). A set of ascorbic acid (AA) standards with concentrations ranging from 10 to 300 µg mL^−1^ was used for calibration. The total antioxidant activity was expressed as the number of grams equivalent to ascorbic acid (µg AAEq g^−1^) [37].

#### 3.4.4. ATR-FTIR

Ulvan functional groups were determined on an attenuated total reflection-Fourier transformation infrared (ATR-FTIR) spectrophotometer (Vertex70, Bruker Instruments, Bremen, Germany). Dried samples were scanned 50 times at wave numbers ranging from 650 to 4000 cm^-1^ with a resolution of 4 cm^−1^. Data were processed with OPUS 8.5 software, and baseline correction and normalization were conducted.

## 4. Conclusions

In this work, the effects of the enzyme type, extraction time, and the application of ultrasound were investigated on the extraction yields and main ulvan physicochemical properties. The combination of enzymatic (Cellulysin 17 h, 300 U g^−1^_Biomass_, pH 5, 40 °C) and ultrasound-assisted extraction (80% amplitude, 40 min) led to the highest ulvan yield extraction (17.92% dw). Further optimization of the enzymatic reaction (e.g., enzyme activity, temperature, simultaneous use of different enzyme types, etc.) represents a potential approach for future improvement of the extraction yields. The characterization of the extracted ulvans showed that low levels of impurities were present, without structural modification of the extracted polysaccharides. In addition, extracted ulvan showed a total antioxidant capacity of up to 197 ± 94.5 g kg^−1^. With a similar essential amino acids content to other vegetal protein sources, the residual biomass after ulvan extraction represents a potential alternative protein source.

Enzyme-assisted extraction is a promising approach for extracting macroalgae biopolymers. However, the cost of certain enzymes can remain a significant bottleneck that could be tackled by enhancing enzyme reuse in several extraction cycles (e.g., enzyme immobilization). A technoeconomic and environmental assessment of ulvan extraction by different approaches (e.g., hot water, EAE, mechanical extraction, etc.) could enable a holistic assessment of the overall performance of the different extraction methods.

The enzyme-assisted extraction of ulvan for the development of biopolymer films aligns with several Sustainable Development Goals, including SDGs #12 and #14. This approach contributes to sustainable production, responsible consumption, climate action, and marine ecosystem preservation.

## Figures and Tables

**Table 1:** Amino acid profile of *Ulva fenestrata* biomass compared to the World Health Organization (WHO) recommendations [17].

Amino Acid	Amino Acid Distribution (% of TAA)	
	*Ulva fenestrata*	WHO Requirement (% EAA in Total Protein)
Aspartic acid	15.1 ± 0.06	
Glutamic acid	12.1 ± 0.09	
Serine	5.86 ± 0.08	
Histidine *	2.74 ± 0.03	1.5
Glycine	6.21 ± 0.04	
Threonine *	5.42 ± 0.05	2.3
Arginine	5.52 ± 0.06	
Alanine	9.59 ± 0.03	
Tyrosine	3.02 ± 0.06	
Valine *	6.29 ± 0.07	3.9
Methionine *	1.72 ± 0.01	1.6
Phenylalanine *	6.38 ± 0.004	3.8
Isoleucine *	4.39 ± 0.07	3
Leucine *	7.39 ± 0.02	5.9
Lysine *	4.57 ± 0.01	4.5
Proline	3.75 ± 0.03	
Total protein content (% dw)	19.8 ± 1.31	
Total essential amino acids (% dw)	38.9 ± 0.04	

* Essential amino acids.

**Table 2:** Average content of total phenolic compounds (TPCs), proteins, and total antioxidant capacity (TAC) in ulvans extracted by enzyme-assisted extraction.

Enzyme	TPCs, g kg^−1^	Proteins, g kg^−1^	TAC, g kg^−1^
Viscozyme L	0.25 ± 0.024	24.5 ± 4.50	131 ± 16.1
Cellulysin	0.16 ± 0.026	62.5 ± 7.05	197 ± 94.5
Neutrase 0.8L	0.13 ± 0.034	130 ± 15	106 ± 21.9
Flavourzyme	0.16 ± 0.023	81.5 ± 29.4	115 ± 40.5

**Table 3:** Wavenumbers and respective functional groups.

Band	Wavenumber (cm^−1^)	Functional Group
a	3500–3000	–OH; N–H
b	2970	C–H
c	2930	C–H
d	1720	C=O
e	1600	C=O; C=C (arom.); –N–H
f	1420	COO^−^
g	1380	–CH_3_
h	1260	S=O
i	1215	C–O; C–N
j	1045	C–O
k	980	C–O–C
l	840	C–O–S

**Table 4:** Total protein content (% dw) and essential amino acids (% dw) in the residual biomass after EAE with Cellulysin and Neutrase 0.8L and U-EAE.

Residual Biomass	Protein Content,% dw	EAA Content,% dw
Cellulysin	26.5	40.6
Neutrase 0.8L	18.3	40.9
U-EAE	23.7	40.4
Raw biomass	19.8 ± 1.31	38.9 ± 0.04

## Data Availability

Not applicable.
